# From harmful Microcystis blooms to multi-functional core-double-shell microsphere bio-hydrochar materials

**DOI:** 10.1038/s41598-017-15696-9

**Published:** 2017-11-13

**Authors:** Lei Bi, Gang Pan

**Affiliations:** 1 Department of Environmental Nano-materials, Research Center for Eco-Environmental Sciences, Chinese Academy of Sciences, Beijing, 100085 China; 2 School of Animal, Rural, and Environmental Sciences, Nottingham Trent University, Nottingham, NG25 0QF UK

## Abstract

Harmful algal blooms (HABs) induced by eutrophication is becoming a serious global environmental problem affecting public health and aquatic ecological sustainability. A novel strategy for the utilization of biomass from HABs was developed by converting the algae cells into hollow mesoporous bio-hydrochar microspheres via hydrothermal carbonization method. The hollow microspheres were used as microreactors and carriers for constructing CaO_2_ core-mesoporous shell-CaO_2_ shell microspheres (OCRMs). The CaO_2_ shells could quickly increase dissolved oxygen to extremely anaerobic water in the initial 40 min until the CaO_2_ shells were consumed. The mesoporous shells continued to act as regulators restricting the release of oxygen from CaO_2_ cores. The oxygen-release time using OCRMs was 7 times longer than when directly using CaO_2_. More interestingly, OCRMs presented a high phosphate removal efficiency (95.6%) and prevented the pH of the solution from rising to high levels in comparison with directly adding CaO_2_ due to the OH^−^ controlled-release effect of OCRMs. The distinct core-double-shell micro/nanostructure endowed the OCRMs with triple functions for oxygen controlled-release, phosphorus removal and less impact on water pH. The study is to explore the possibility to prepare smarter bio-hydrochar materials by utilizing algal blooms.

## Introduction

Eutrophication promotes the growth of harmful algal blooms (HABs), a process that has been observed in freshwaters worldwide^1^. HABs often results in the release of noxious substances and severe depletion of dissolved oxygen (DO), which threatens the drinking-water security of nearby residents and aquatic communities^2^. The removal and harvesting of HABs from freshwaters is becoming a practical measure in some places. For instance, more than 200,000 tons of fresh cyanobacteria are salvaged from Taihu Lake, China every year^3^. It has been reported that the main species of cyanobacteria blooms in Lake Taihu are dominated by Microcystis species^4^. However, the task of handling the skimmed cyanobacterial biomass is becoming a heavy burden for local governments. Without appropriate treatment, cyanobacterial biomass can cause serious secondary environmental pollution^5^. Therefore, several methods to utilize cyanobacteria have been developed. The production of biogas and compost from salvaged cyanobacteria potentially allows massive quantities of cyanobacterial biomass to be eliminated by anaerobic digestion. However, due to the low C/N ratio and high moisture content of cyanobacteria, the addition of an external carbon source and the dehydration are necessary, which inevitably increase the cost and energy consumption^5–7^. Carbon materials, such as activated carbon for use as an adsorbent^8^, carbon dots for *in vitro* imaging^9^, and hard carbon material for sodium-ion batteries^10^, have also been prepared from cyanobacteria, but high-temperature calcination and desiccation are often used. Existing cyanobacterial utilization strategies are mainly focused on the breakdown and conversion of cyanobacterial biomass into fertilizer, fuel or carbon materials, and thus neglect the preservation and exploitation of natural spherical structure of Microcystis. The structural characteristics of materials determine their functions. Hence, the development of new strategies for utilizing Microcystis, based on their high moisture content and morphological features, to produce high-value multifunctional products, such as hollow porous microspheres, may bring new material, environmental and economic benefits.

Multifunctional hollow porous microspheres, have been widely used in the fields of controlled release^11^, catalysis^12^, adsorption^13,14^, drug delivery^15,16^, electrode material^17^, sensors^18^ and microreactors^19^. Recently, yeast^20,21^, pollen^22^, spores^23,24^ and chlorella^25^ have been converted into hollow porous microspheres by directly using the natural spherical morphology and components of these micro-organisms via hydrothermal carbonization (HTC), solvent extraction, or carbonization method. This strategy has been defined as microorganism self-template method, which is different from conventional biotemplating method^26,27^. Microorganism self-template method has its own advantages, such as without using templates, simpler procedures and reducing the consumption of harmful chemical agents^27^. However, yeast, pollen or chlorella are industry products or popular tonic food with relative limited yield and high prices. In contrast, harvesting HABs is becoming feasible as cost-effective technologies developed^28^ and HABs is conventionally treated as hazardous waste with little values. Therefore, the use of cyanobacteria as the raw material for the production of hollow porous microspheres is preferable in terms of cost. In addition, due to its natural spherical structure, tough cell wall and fragile protoplast, cyanobacteria is suitable for constructing hollow microspheres provided appropriate treatment measures are developed. However, the main chemical constituent of the cell walls of cyanobacteria are peptidoglycan and lipopolysaccharides which are different from that of yeast, pollen or chlorella^29^. Hence, it remains unclear which treatment method is most suitable for the conversion of cyanobacteria into hollow porous microspheres.

DO is important to water quality and natural aquatic ecosystem. Water anaerobism can cause the release of phosphorus (P) from sediments^30^. P has been regarded as the key limiting factor for HABs formation in freshwaters^31^. Calcium peroxide (CaO_2_) is widely known as a highly efficient oxygen-releasing agent that can rapidly restore depleted DO levels and has been utilized to remediate polluted water bodies^32^. However, the direct use of CaO_2_ powder often results in the burst release of oxygen and a short-term rise in the pH of natural water bodies, which not only decrease the oxygen utilization rate but also threatens the aquatic ecosystem^33^. Hollow porous microspheres, owing to their inner cavities for offering a space for chemical reactions to take place or for encapsulating guest materials, and the permeable shells for modulating mass transport, are suitable for the fabrication of controlled-release materials^34^. Therefore, loading CaO_2_ into hollow porous microspheres to prepare oxygen controlled-release microspheres (OCRMs) may overcome the shortcomings of the direct use of CaO_2_. To the best of our knowledge, there has been little study of the fabrication of hollow porous microspheres using Microcystis or of the usage of prepared hollow porous microspheres as microreactors and carriers for the synthesis of OCRMs.

In this paper, we attempt to develop a novel strategy for the utilization of high-moisture and harmful Microcystis biomass for producing bio-hydrochar hollow mesoporous microspheres (HMMs). We studied appropriate methods for preserving and utilizing the natural spherical morphology and components of cyanobacterial cell walls and converting Microcystis into OCRMs by using Microcystis-derived hollow porous microspheres as microreactors and carriers. The resulting OCRMs will be tested for their oxygen-releasing properties and phosphate-removal ability in water. The objective of the study is to explore the method and mechanism of utilizing harmful algae for producing multifunctional advanced materials.

## Results and Discussion

### Preparation and Characterization of HMMs and OCRMs

**Figure 1:**
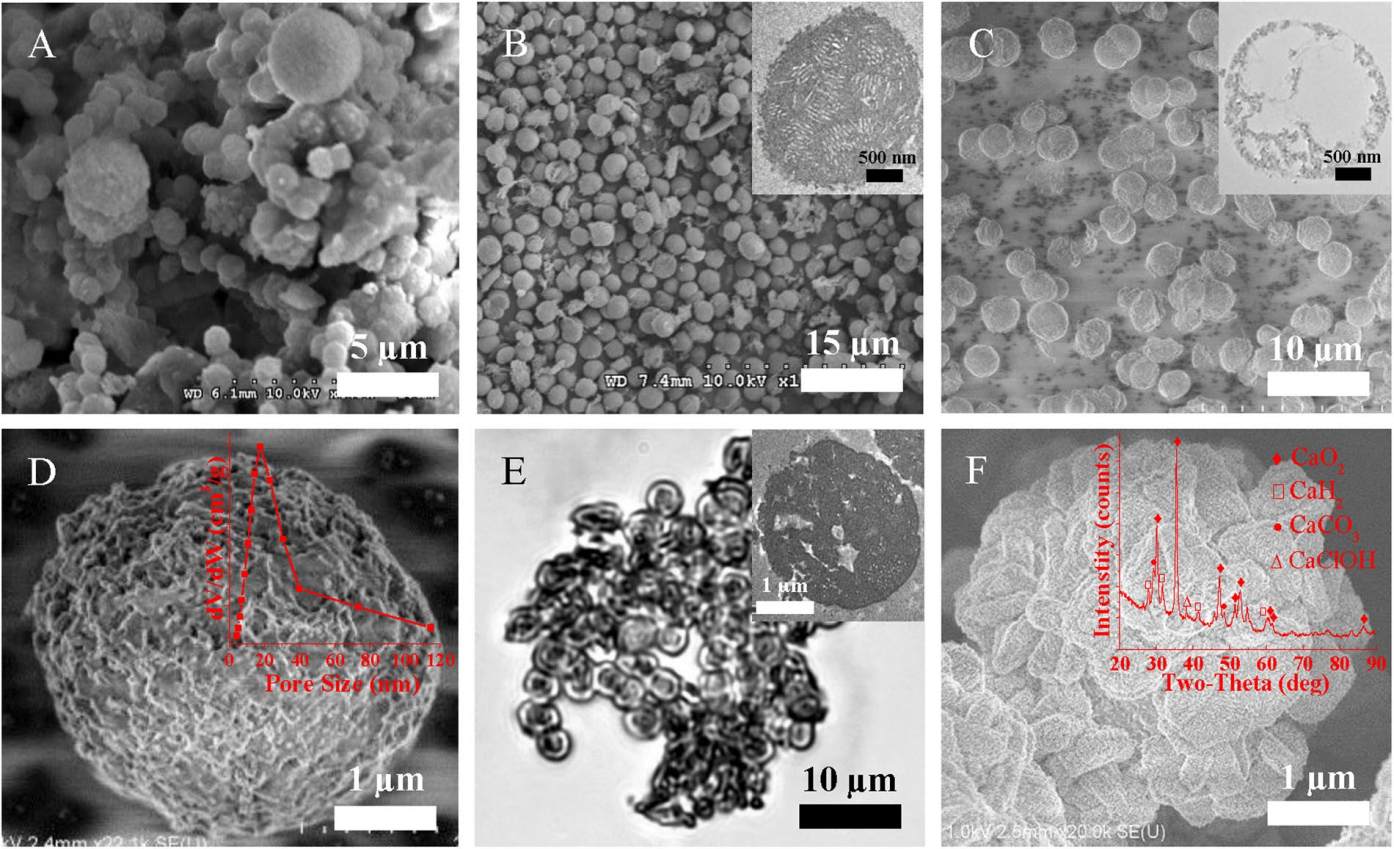
SEM images of (**A**) CM-2 and (**B**) CM-4; FESEM images of (**C**) CM-5 in wide range and (**D**) CM-5 at high magnification; OM image of (**E**) product obtained by dispersing OCRMs into aqueous solution; FESEM image of (**F**) OCRMs at high magnification. The insets in (**B**), (**C**), (**D**), (**E**) and (**F**) are TEM images of ultrathin sections of a single CM-4 and of a single CM-5, pore size distribution in CM-5, TEM image of an ultrathin section of single OCRMs and XRD pattern of OCRMs, respectively.

Several synthetic strategies were conducted to obtain HMMs by treating fresh cyanobacteria as the raw material, the codes used to identify the samples are indicated in Supplementary Table S1. Supplementary Fig. S1 shows that the product of oven-treating the cyanobacteria at 105 °C for 8 h did not exhibit the morphology of single, distributed microspheres, but rather, all of the cells conglutinated together. Supplementary Fig. S2 shows that the product of carbonization treatment of the cyanobacteria (fixed with 1.5% glutaraldehyde) in argon at 700 °C for 4 h displayed a morphology based on large blocks with no discernible sphere-like structure, indicating that the high temperature had completely destroyed the cell walls. However, it can been seen from Fig. 1A that the hydrothermal treatment of the cyanobacteria at 200 °C for 8 h (CM-2) preserved the microsphere morphology of the cyanobacterial cells to a certain extent, although the product exhibited irregularly spherical structures, some of which presented conglutination. Supplementary Table S1 lists the structural properties of the products of hydrothermal treatment of the cyanobacteria for different holding times. The data show that the average pore size, pore volume and Brunauer-Emmett-Teller (BET) surface area first increased and then decreased as the holding time was increased from 0 to 10 h, and reached a maximum at 8 h. Thus, 8 h is considered to be the optimal hydrothermal reaction time. To avoid the destruction of the microsphere structure, glutaraldehyde was used as a protecting agent in one sample (CM-4) to enhance the strength of the cell walls. Figure 1B shows that this product exhibited intact spherical structures after the addition of glutaraldehyde. However, the ultrathin section of CM-4 shown in the inset of Fig. 1B suggests that the product did not exhibit hollow structure due to the fixation of the protoplasts of the cyanobacteria by the glutaraldehyde. As shown in Supplementary Table S1, the addition of glutaraldehyde decreased the values of pore volume and BET surface area from 0.061 cm^3^/g and 20.44 cm^2^/g, respectively (for CM-2) to 0.025 cm^3^/g and 8.44 cm^2^/g, respectively (for CM-4). In order to obtain hollow structure, cyanobacteria were pretreated with sodium dodecyl sulfate (SDS) to destroy and extract the protoplast, and glutaraldehyde was added to protect the cell walls (CM-5). Figure 1C is the wide-range cold field-emission scanning electron microscope (FESEM) image of CM-5, in which the microspheres present good monodispersity and intact spherical structures. The ultrathin section of CM-5 shown in the inset of Fig. 1C confirms the existence of large hollow structure in microsphere. Furthermore, the high-magnification image in Fig. 1D reveals the presence of numerous pores in the shell, with an average pore size of 18.08 nm. Furthermore, as shown in Supplementary Table S1, after pretreatment of the cyanobacteria with SDS, the average pore size, pore volume and BET surface area of CM-5 were significantly improved in comparison with CM-2 and CM-4. However, the treatment without adding glutaraldehyde (Microcystis were pretreated with SDS) shows that the sphere form of cell walls have been destroyed after the treatment of HTC method (see Supplementary Fig. S3-A and B). Glutaraldehyde can improve the hydrolysis resistant of the cell walls and preserve the sphere form of the Microcystis cells. The powder of CM-5 showed a brown color which presents the feathers of hydrochar (see Supplementary Fig. S4). Therefore, the morphological and structural characterization of the products showed that HMMs had been successfully fabricated via the hydrothermal treatment of fresh cyanobacteria, and CM-5 was chosen as the optimal sample for the fabrication of OCRMs.

The preparation of the OCRMs was based on the coprecipitation of H_2_O_2_ and CaCl_2_ in NH_4_OH solution. Figure 1E displays an OM image of the OCRMs in aqueous solution, in which almost every cavity of CM-5 has been loaded with particles and the sample exhibits a core @ shell structure. Moreover, in comparison with the hollow structure of CM-5 in the inset of Fig. 1C, the ultrathin section of the OCRMs shown in the inset of Fig. 1E further demonstrates that particles had been successfully encapsulated into CM-5. Meanwhile, the rough and irregular surface of the OCRMs in the FESEM image (Fig. 1F) shows that part of the coprecipitated crystals also grew on the external surfaces of the HMMs. The low-magnification FESEM image in Fig. S3 suggests that most of the CM-5 microspheres were covered with crystals of this kind, and a certain degree of aggregation occurred on the OCRMs after coprecipitation. XRD analysis was conducted to identify the composition of the OCRMs. As shown in the inset of Fig. 1F, the representative diffraction peaks at the positions of 30.1°, 35.5°, 47.2°, 51.6°, 52.9°, 60.6° and 86.7° matched well with the standard PDF card of CaO_2_ (# 03-0865), confirming that CaO_2_ was formed in this procedure. Meanwhile, the representative diffraction peaks of CaH_2_, CaCO_3_ and CaClOH were also identified in the product, being common impurities in the fabrication of CaO_2_^35,36^. In summary, these results confirm that CaO_2_ had been successfully encapsulated into the HMMs (CM-5) and that part of the CaO_2_ covered the surface of the microspheres, which exhibited a distinct CaO_2_ core @ mesoporous shell @ CaO_2_ shell structure.

### Characterization and analysis of HMMs

To explore the formation mechanism of the HMMs, chemical element analysis and Fourier transform infrared (FTIR) spectrometry were carried out. The results in Table 1 indicate that the C content increased from 45.12 wt.% in CM-0 to 56.91 wt.% in CM-2. At the same time, there was a reduction of the H, O and N contents and the H/C and H/O ratios in CM-2, which is consistent with the carbonization process^37^. It should be noted that, after glutaraldehyde was added, the C content in CM-5 was 66.41 wt.% which was higher than that of CM-2. The notable increase of the C content in CM-5 may be attributable to the participation of glutaraldehyde in the formation of the HMMs, which will be discussed in relation to the FTIR spectral analysis. Moreover, Table 1 shows that the N content decreased from 11.36 wt.% in CM-0 to 7.43 wt.% in CM-2. As is known, most of the N content in cyanobacteria is found in the proteins, including a minor fraction found in the peptidoglycan of the cell walls^29^. The decline of the N content in CM-2 implied that N was lost in the form of liquid or volatile decomposition products^38^. Furthermore, the pretreatment of the raw cyanobacteria with SDS further reduced the N content to 2.89 wt.% in CM-5, which indicated that most of the protein inside the cyanobacteria had been extracted. SDS is a highly efficient surfactant that is known to destroy cell membranes and dissolve lipids and proteins^39^. Therefore, the marked reduction of the N content in CM-5 provided indirect evidence of the removal of protoplasm from the cyanobacteria.

**Table 1:** Elemental composition of the CM-0, CM-2 and CM-5.

Sample	Chemical composition					
	C(wt.%)	H(wt.%)	O(wt.%)	N(wt.%)	H/C(at.)	O/C(at.)
CM-0	45.12	6.44	34.13	11.36	1.713	0.567
CM-2	56.91	5.81	27.02	7.43	1.225	0.356
CM-5	66.41	5.19	24.04	2.89	0.937	0.271

**Figure 2:**
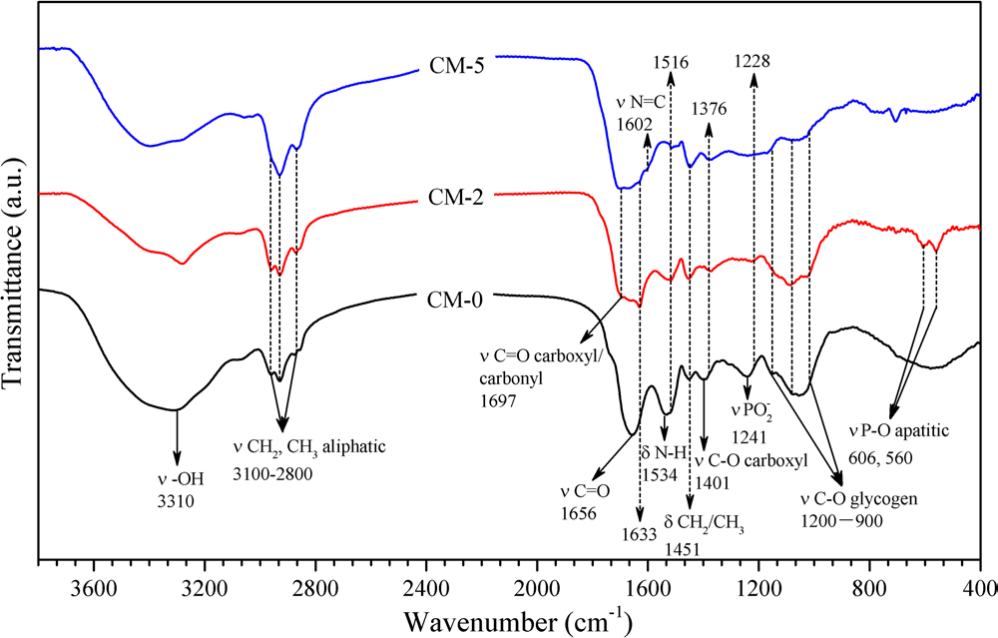
FTIR spectra of the CM-0, CM-2 and CM-5.

The FTIR spectra in Fig. 2 shows that the peaks of the amide I, amide II and carboxyl bands^40^ shifted from 1656, 1534 and 1401 cm^−1^ in CM-0 to 1633, 1516 and 1376 cm^−1^ in CM-2. In addition, the peak intensity of each of the red-shifted bands in CM-2 became weaker than that of CM-0. These results suggest that the protein structure was destroyed by the high temperature^41^. Moreover, new bands were observed, including two at 606 and 560 cm^−1^, which originated from the P-O stretching vibration of apatitic PO_4_^3−^ ^42^. In contrast, the band intensity in the range 1200–1250 cm^−1^, characteristic of PO_2_^−^ stretching vibrations from the phosphodiester backbone of nucleic acid^39^, weakened drastically, which implies that the hydrothermal treatment induced the mineralization of DNA and RNA inside the cyanobacteria^42^. Furthermore, the bands in the range 900–1200 cm^−1^ and the band centered at 3310 cm^−1^ in CM-0 can be assigned to C-O-C stretching vibrations of polysaccharides and O-H stretching vibrations of hydroxyl or carboxyl groups, respectively^40^. However, the decrease (in relation to CM-0) in the intensity of the bands at 900–1200 cm^−1^ and 3100–3700 cm^−1^, and the new C=O stretching vibration bands at 1697 cm^−1^ appearing in CM-2, indicated that dehydration occurred during the hydrothermal carbonization of the cyanobacteria^43,44^. This result was consistent with the evolution of the H/C and O/C atomic ratios. Therefore, the C=O stretching vibration bands can be assigned to a carboxyl group and/or a carbonyl group formed by the dehydration of a hydroxyl group^45^, the latter being abundant in cyanobacteria. After pretreatment with SDS, the amide I, amide II and PO_2_^−^ bands in CM-5 almost disappeared, strong evidence that most of the protein and nucleic acid inside the cyanobacteria had been successfully removed. In addition, the new shoulder peak at 1602 cm^−1^ in CM-5, which was not present in CM-2, is assigned to an imine bond (N=C), which indicates that a Schiff base had been formed by the reaction between the amines in the cell walls and the aldehyde in glutaraldehyde^46^. This incorporation of glutaraldehyde into the cell walls by chemical reaction is the most likely source of the increased C content in CM-5.

In summary, by structural characterization, elemental analysis and FTIR spectroscopy, we have confirmed that hydrothermal treatment improved the structural properties of the products and induced the conversion of cyanobacteria to hydrochar. By pretreatment with SDS and the addition of glutaraldehyde, the final product exhibited large cavities in the microspheres, abundant mesopores in the shells and high thermolysis-resistance and structural stability, desirable properties for microreactors and carriers for constructing OCRMs.

### Oxygen-releasing properties of OCRMs

**Figure 3:**
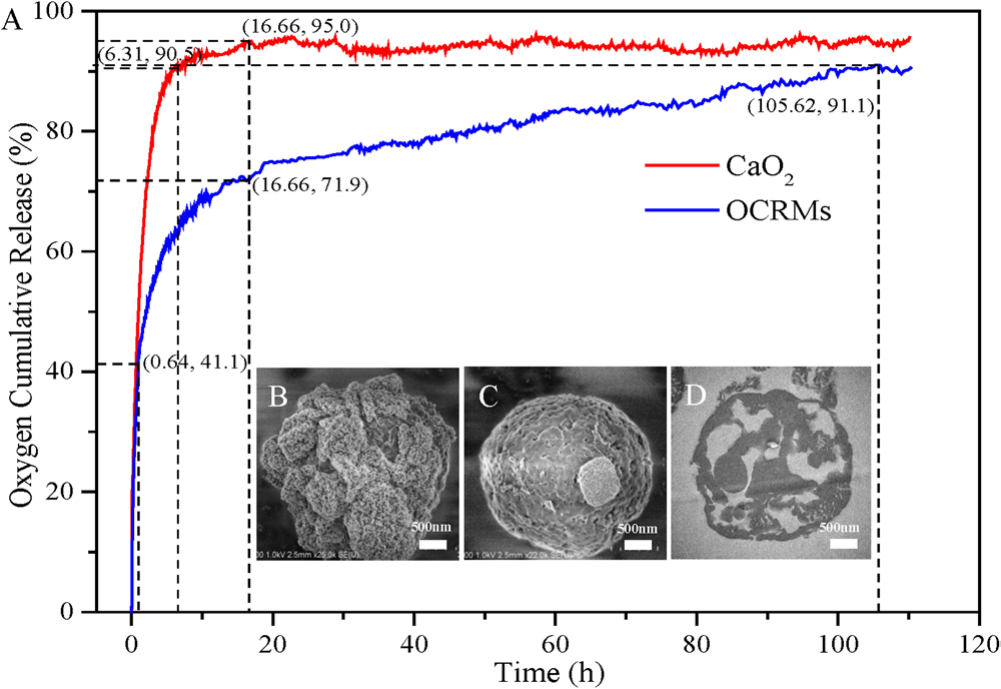
(**A**) Shows the oxygen cumulative release profiles obtained by dispersing CaO_2_ and oxygen controlled-release microspheres (OCRMs) in anaerobic water in a closed system at 25 °C; (**B**), (**C**) and (**D**) are FESEM images and a TEM image of an ultrathin section obtained from the product of the OCRMs reacting with water at 0, 0.64 and 16.66 h, respectively. The numbers in the brackets are data points (hour, percentage) at key reaction stages.

The oxygen-releasing performance of the OCRMs was tested by separately dispersing powder samples (200 mesh) of the OCRMs and of CaO_2_ reagent with the same available CaO_2_ content in anaerobic water, with the experiments performed in a closed system at 25 °C to prevent gas exchange. Figure 3A shows that both the OCRMs and the CaO_2_ presented fast oxygen release, and the oxygen cumulative release amount for both samples increased from 0 to 41.1% during the initial 0.64 h. However, while the oxygen release rate of the OCRMs began to slow after 0.64 h, the CaO_2_ treatment maintained a high oxygen release rate until 6.31 h. The oxygen cumulative release amount of the CaO_2_ treatment arrived at its maximum value of 95.0% at 16.66 h and subsequently plateaued, but the OCRMs treatment maintained its slow release of oxygen and the oxygen cumulative release amount of the latter was three-fourths that of the former at this point in time. The OCRMs treatment finally reached its maximum oxygen cumulative release amount of 91.1% after 105.62 h, meaning that the OCRMs had maintained the release of oxygen for nearly 7 times longer than CaO_2_. As is known, CaO_2_ can dissolve in water to produce oxygen (equation (1)). It can be seen that the CaO_2_ shell on the surface of the OCRMs in Fig. 3B

1 $${{\rm{CaO}}}_{{\rm{2}}}({\rm{s}})+{{\rm{H}}}_{{\rm{2}}}{\rm{O}}={{\rm{Ca}}}^{{\rm{2}}+}(\mathrm{aq})+2{{\rm{OH}}}^{-}({\rm{aq}})+0.5{{\rm{O}}}_{{\rm{2}}}({\rm{g}})$$

mostly disappeared after the dispersal of the OCRMs in water at 0.64 h in Fig. 3C. Furthermore, when the CaO_2_ treatment reached its maximum oxygen cumulative release amount at 16.66 h, the OCRMs treatment had released just 71.9% of its eventual oxygen cumulative release amount. From the ultrathin section in Fig. 3D, showing the product of the reaction of the OCRMs with water at 16.66 h, it can be estimated that nearly 30% CaO_2_ remained within the interior of the OCRMs. Therefore, by considering both the oxygen cumulative release amount profiles and the morphological changes of the OCRMs, the following mechanism is proposed for the controlled release of oxygen by the OCRMs. The initial burst release of oxygen from the OCRMs was attributed to the rapid decomposition of the external CaO_2_ shells by reaction with water; however, once the external CaO_2_ part of the OCRM shells was depleted, the remaining mesoporous shells of the OCRMs acted as a barrier to block the entrance of water and the release of oxygen, which significantly decreased the oxygen release rate and in turn prolonged the oxygen release duration of the OCRMs. Therefore, the OCRMs not only rapidly improved the DO of anaerobic water bodies in a short period but also considerably extended the oxygen release time of CaO_2_.

### Phosphate-removal ability of OCRMs and their effect on aquatic pH

**Figure 4:**
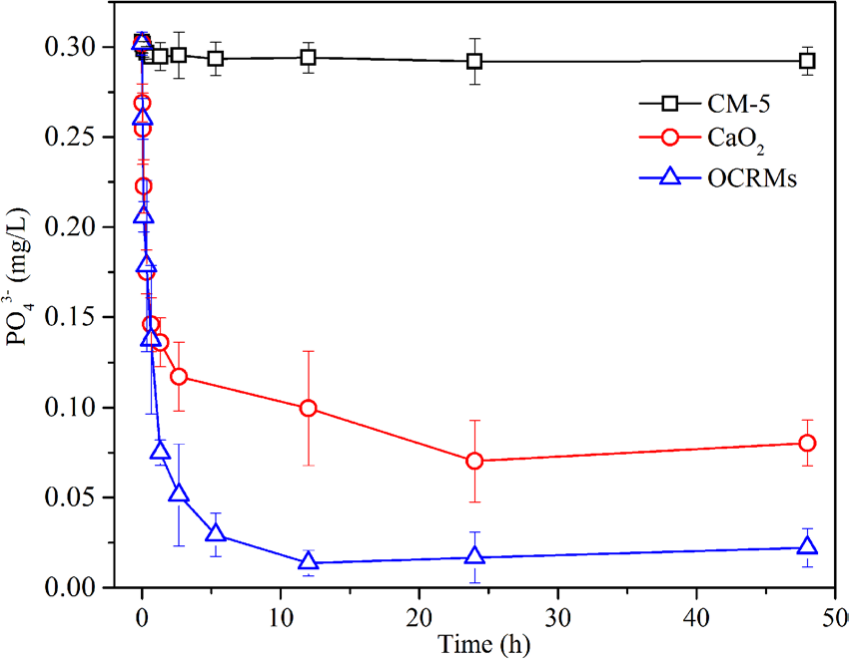
Phosphate removal by oxygen controlled-release microspheres (OCRMs) and CaO_2_ with time in the experiment. The initial phosphate concentration is around 0.3 mg/L (pH 7.2). Both OCRMs and CaO_2_ have the same available CaO_2_ content.

The phosphate-removal ability of the OCRMs and their effect on pH were studied in an open system. The lowest dosages of the CaO_2_ reagent and of the OCRMs with the same available CaO_2_ content were used in order to ensure that the DO of the anaerobic water could be increased to approximately 8 mg/L. As shown in Fig. 4, the initial phosphate concentration of approximately 0.303 mg/L decreased rapidly to 0.146 mg/L in the CaO_2_ treatment and 0.137 mg/L in the OCRMs treatment during the initial 0.67 h. Subsequently, the phosphate content in the CaO_2_ treatment decreased more slowly than that of the OCRMs treatment. After 12 h, the phosphate concentration in the OCRMs treatment stabilized at its minimum value of 0.013 mg/L, corresponding to a phosphate removal efficiency of 95.7%. In contrast, the CaO_2_ treatment reached its minimum phosphate concentration of 0.070 mg/L after 24 h, with a phosphate removal efficiency of 76.8%. Moreover, in the control experiment using CM-5 microspheres without a loading of CaO_2_, it was found that the bare HMMs had little effect on the phosphate concentration.

**Figure 5:**
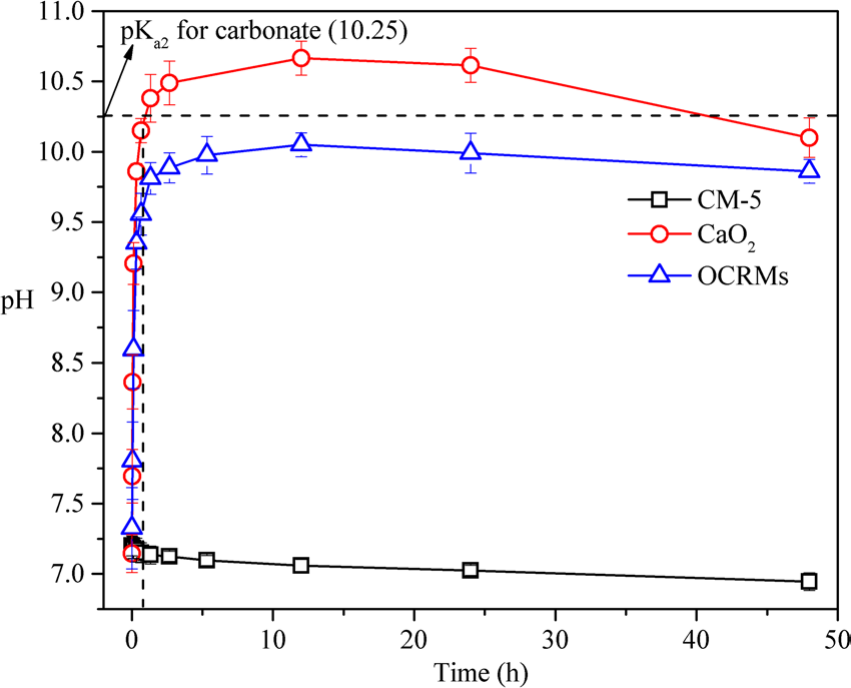
pH change of oxygen controlled-release microspheres (OCRMs) and CaO_2_ with time in the experiment. The initial pH is around 7.2 (with 0.3 mg/L phosphate). Both OCRMs and CaO_2_ have the same available CaO_2_ content.

As shown in Fig. 5, before the addition of OCRMs and CaO_2,_ the pH values of the solutions were around 7.20. After adding the sample powders, the solution pH rapidly increased to 9.56 in the OCRMs treatment and 10.15 in the CaO_2_ treatment, respectively, in the initial 0.67 h, due to the production of OH^−^. Subsequently, the pH in the OCRMs treatment increased more slowly than that of the CaO_2_ treatment. After 12 h, the peak value of pH for OCRMs treatment was 0.7 lower than that of the CaO_2_ treatment. It is well known that CO_2_ in air can be dissolved in natural water to form H_2_CO_3_ under acidic and neutral conditions, and H_2_CO_3_ can neutralize OH^−^ to produce HCO_3_^−^ and CO_3_^2−^ in basic conditions. As the reaction continued, CO_2_ in the air continually dissolved and neutralized OH^−^ in the solutions, causing the pH to decline in both treatments. Adding CM-5 alone has little effect on the pH of the solutions (Fig. 5). In comparison with directly addition of CaO_2_, the encapsulation of CaO_2_ in CM-5 prevented the pH of the solution from rising to high levels, due to the synergy of the controlled release of OH^−^ from the OCRMs and the neutralizing effect of acidic CO_2_. Therefore, OCRMs presented the ability to inhibit the pH of water to high levels to some extent.

It has been reported that, HPO_4_^2−^, PO_4_^3−^ and CO_3_^2−^ can bond Ca^2+^ to form the precipitations of Ca-phosphate minerals and calcium carbonate^47^. HPO_4_^2−^ is the dominant ionic form of phosphate in the pH range of 8–11^48^. HPO_4_^2−^ may be a major form of phosphate in addition to PO_4_^3−^ in the two treatments. CO_3_^2−^ is the dominant ionic form of carbonate, when the pH of solution is above the pK_a2_ value for carbonate 10.25^49^. The pH value in the CaO_2_ treatment was higher than 10.25 from 1.33 to 40 h, and the CaHPO_4_ has a higher solubility (K_sp_ = 1.00 × 10^−7^) than CaCO_3_ (K_sp_ = 2.80 × 10^−9^)^50^. It can be inferred that part of Ca^2+^ may be consumed by the CO_3_^2−^ in the CaO_2_ treatment during this time. In contrast, the maximum pH value in the OCRMs treatment was 10.05, which is less than the pK_a2_ value for carbonate. Therefore, the regulation of the solution pH by the OCRMs prevented the generation of large quantities of CO_3_^2−^ ions competing to bind Ca^2+^ in the phosphate removal experiment and thus resulted in the OCRMs achieving much higher phosphate removal efficiency than CaO_2_.

## Conclusions

In this paper, Microcystis from HABs was converted into multifunctional bio-hydrochar microspheres materials (HMMs and OCRMs). HMMs are good candidates as microreactors and carriers for constructing OCRMs. Due to the distinct core-double-shell micro/nanostructure, OCRMs not only self-regulated the oxygen release rate, but also presented a high phosphate removal efficiency and less change on water pH in comparison with directly adding CaO_2_ powder. We have reported the effects of OCRMs on oxygen release and phosphorus removal in waters, further studies may be triggered to test the smart algae bio-hydrochar in remediating metal or organic pollutants in soil and for safe agricultural production.

### Data availability statement

All data generated or analyzed during this study are included in this article (and its Supplementary Information files).

## Experimental

### Preparation of hollow mesoporous microspheres (HMMs)

The fresh cyanobacteria used in our experiment were collected from Lake Taihu Wuxi, China in July 2012. Lab analysis indicated that it was dominated by Microcystis species (see Supplementary Fig. S6), which agreed with literature^4^. Oven-dried cyanobacteria, denoted CM-0, were obtained by centrifugation and drying fresh cyanobacteria in an oven at 105 °C for 8 h. Carbonized cyanobacteria were obtained by carbonizing freeze-dried cyanobacteria, which had previously been fixed with 1.5% glutaraldehyde, at 700 °C in an argon-protected furnace for 4 h. The hydrothermal carbonization (HTC) of cyanobacteria was performed according to the following procedures: 80 mL fresh cyanobacteria slurry (0.7 g in dry weight) was transferred to a stainless steel autoclave, heated to 200 °C and maintained at this temperature for 6, 8 or 10 h, to create samples denoted CM-1, CM-2 and CM-3, respectively. After the reaction, the autoclave was allowed to cool to room temperature, and the resulting solid products were recovered by centrifugation and washed with distilled water several times. Then, each sample was dried at 105 °C for 4 h for further analysis. A sample supplemented with the addition of glutaraldehyde was also prepared by adding 2.4 mL 50% glutaraldehyde to 80 mL fresh cyanobacteria slurry and stirred for an additional 5 min at 25 °C. After that, the mixture was transferred to a stainless steel autoclave for HTC treatment at 200 °C for 8 h, and the resulting sample was denoted CM-4. To create a sodium dodecyl sulfate (SDS)-pretreated sample, 80 mL fresh cyanobacteria slurry was dispersed in 320 mL 2.5% (w/v) SDS alkaline solution and stirred for 2 h at 95 °C. Then, the mixture was centrifuged and washed with distilled water several times. The product was diluted to 80 mL in distilled water, 2.4 mL 50% glutaraldehyde was added to the mixture, and it was stirred for an additional 5 min at 25 °C. After that, the product was transferred to a stainless steel autoclave for HTC treatment at 200 °C for 8 h, and the resulting sample was denoted CM-5. The experimental conditions (temperature, time and additive) used in these experiments are listed in Supplementary Table S1. The codes used to identify the samples are also indicated in this table.

### Fabrication of OCRMs

1 g CM-5 was dispersed in 20 mL 70 g/L CaCl_2_ solution and stirred at room temperature for 1 h. After isolation by centrifugation, the solid product was washed once with deionized water and then homogeneously dispersed in cold 20 mL 30% H_2_O_2_. Under continuous stirring, 10 mL ammonia solution (1 M) was added to the stirred mixture at a rate of 20 drops per minute. After stirring for 1 h, the yellow solid was vacuum-filtered and fully washed with deionized water. Finally, the product was transferred to an oven, and maintained at 140 °C for 2 h. After cooling to room temperature, OCRMs powder was obtained. The available content of calcium superoxide in the OCRMs was analyzed by the iodine quantity method^51^.

### Oxygen-releasing properties of OCRMs

Oxygen release from the OCRMs was quantified using a dissolved-oxygen probe in a closed system. Briefly, 0.0668 g OCRMs (53.6% purity, 200 mesh) was added to a 1000 mL round-bottom flask, which was then filled with 1000 mL hypoxic distilled water (DO: 0.05 mg/L, flushed with nitrogen at 25 °C), following which the oxygen probe was placed into the flask and sealed with a rubber stopper to prevent gas exchange. Finally, the flask was incubated in a magnetic-stirring water bath at 25 °C, and stirred at 150 rpm. Data on the DO were automatically collected at intervals of 1 min using a JPSJ-605 real-time DO monitoring system (Rex, China). To perform a control experiment with the same available content of CaO_2_ as that provided by the OCRMs, 0.0527 g CaO_2_ reagent (68.0% purity, 200 mesh) was added to a flask, and all the other experimental conditions were kept the same as in the OCRMs treatment.

### Phosphate-removal ability of OCRMs and their effect on aquatic pH

The real eutrophic lake-water used in this study was taken from Taihu Lake; coarse particles and algal cells were removed using filter paper before use. To test the phosphate-removal efficiency of the OCRMs in severely eutrophic water, a certain amount of 100 mg/L phosphate solution (NaH_2_PO_4_) was added to the lake-water to ensure an initial phosphate concentration of around 0.3 mg/L and a pH of 7.2. Then, 0.0668 g OCRMs (53.6% purity, 200 mesh) or 0.0527 g CaO_2_ reagent (68.0% purity, 200 mesh) was added to 1 L lake-water, and the beaker containing the lake-water was incubated in a magnetic-stirring water bath at 25 °C and stirred at 150 rpm. Water samples were collected at pre-determined intervals of time and filtered with a 0.45 μm filter for phosphate analysis. Meanwhile, the pH level was recorded with a pH meter at the same points in time. In addition, 0.1000 g HMMs (200 mesh) was added to the lake-water to evaluate the effect of the HMMs on the phosphate concentration and the pH. The experiments were repeated three times.

### Characterization and analysis methods

Scanning electron microscopy (SEM), cold field-emission scanning electron microscopy (FESEM) and optical microscopy (OM) microphotographs were obtained with an S-3000N (Hitachi, Japan) at 10.0 kV, an SU8020 (Hitachi, Japan) at 1.0 kV and a BA210 (Motic, China), respectively. Pore size, pore volume and surface area were determined by measuring nitrogen (N_2_) adsorption/desorption isotherms at −196 °C on an ASAP 2020 apparatus (Micrometrics, USA). The data on the N_2_ isotherms were analyzed to calculate the specific surface area based on the Brunauer–Emmett–Teller (BET) method. The pore volume was determined from the amount of nitrogen adsorbed at a relative pressure (P/P_0_) of 0.95. The average pore width was calculated from the equation proposed by Stoeckli and Ballerini^52^. The carbon (C), hydrogen (H), oxygen (O) and nitrogen (N) contents of the samples were analyzed using an elementar (Vario EL III, Germany). The samples were scanned over 500–4000 cm^−1^ using a Nicolet 8700 (Thermo-Fisher, USA) Fourier transform infrared (FTIR) spectrometer at a resolution of 4 cm^−1^. Powder X-ray diffraction was performed on an X’Pert Pro MPD X-ray diffractometer (Philips, the Netherlands) with Cu-Kα radiation at 40 kV and 40 mA with a scanning rate of 5° min^−1^. The phosphate concentrations in the water samples were analyzed using molybdenum blue colorimetry.

## Electronic supplementary material

Supplementary Information
