# Supplementary material for: From harmful Microcystis blooms to multi-functional core-double-shell microsphere bio-hydrochar materials
Source: Sci Rep. 2017 Nov 13;7:15477. doi: 10.1038/s41598-017-15696-9 (PMC5684341; doi:10.1038/s41598-017-15696-9)
Supplement: Supplementary file 1 — Supplementary Information [file 41598_2017_15696_MOESM1_ESM.pdf]

Supporting Information

From harmful Microcystis blooms to multi-functional core-double-shell microsphere bio-hydrochar materials

Lei Bi[1] and Gang Pan[1*, 2]

1. Department of Environmental Nano-materials, Research Center for Eco-Environmental Sciences, Chinese Academy of Sciences, Beijing 100085, China

2. School of Animal, Rural, and Environmental Sciences, Nottingham Trent University, NG25 0QF, UK

*Corresponding author: Gang Pan; phone: +86-10-62849686; fax: +86-10-62849686; e-mail: gpan@rcees.ac.cn

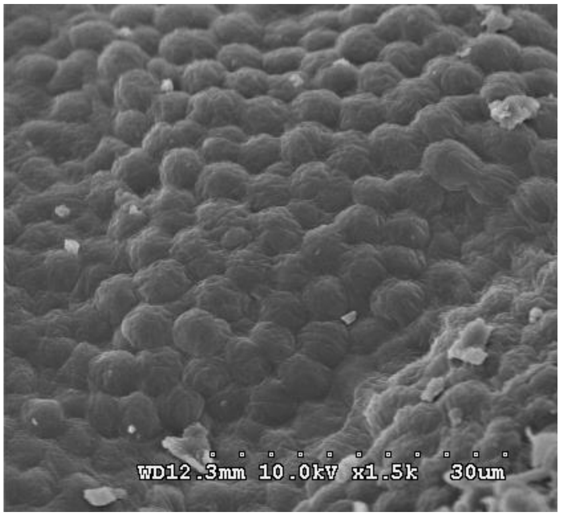

Figure S1. SEM image of product obtained from oven-treating cyanobacteria at 105 ^{o}C for 8 h (CM-0)

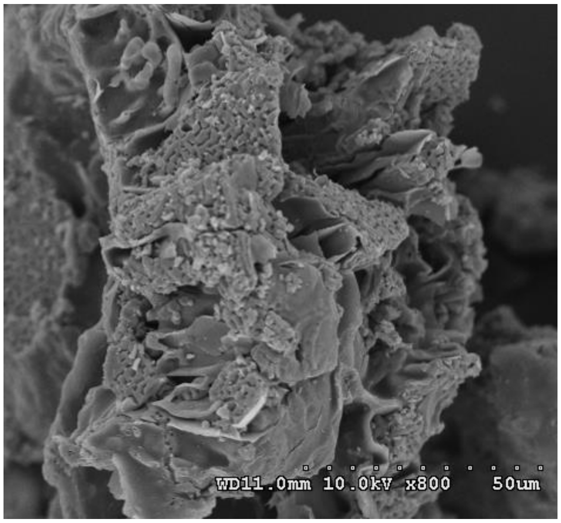

Figure S2. SEM image of product obtained from carbonization treatment of the cyanobacteria (fixed with 1.5% glutaraldehyde) in argon at 700 ℃ for 4 h

Table S1. Synthesis conditions and textural properties of the products

Sample	Fabrication conditions				L_0[c]	V_P[d]	S_{BET}[e]
	T (℃)	T (h)	SDS[a]	GD[b]	(nm)	(cm^3/g)	(m^2/g)
CM-0	105	8	－	－	7.18	0.014	6.79
CM-1	200	6	－	－	8.17	0.018	10.24
CM-2	200	8	－	－	12.00	0.061	20.44
CM-3	200	10	－	－	11.12	0.042	15.21
CM-4	200	8	－	＋	11.71	0.025	8.44
CM-5	200	8	＋	＋	18.08	0.130	28.66

[a] Pretreatment with SDS
[b] Glutaraldehyde
[c] Average pore width was calculated by means of the Stoeckli-Ballerini equation
[d] Pore volume was determined at a relative pressure (P/P_0) of 0.95
[e] BET surface area

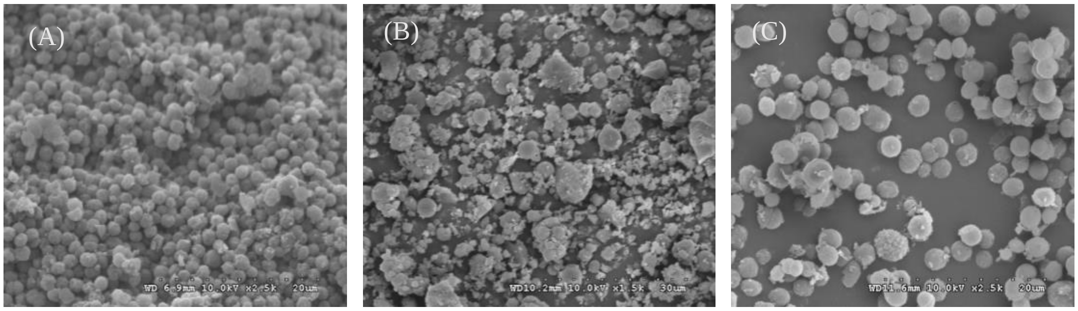

Figure S3 (A), (B) and (C) (A), (B) and (C) are the SEM images of products obtained by pretreating Microcystis with SDS at 105 ℃ (CM-6), HTC treatment of CM-6 without addding glutaraldehyde (CM-7), and HTC treatment of CM-6 with addition of glutaraldehyde (CM-5), respectively.

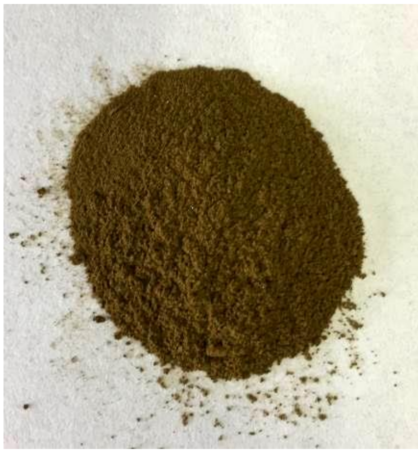

Figure S4. the photograph of the hydrochar powder of HMMs (CM-5)

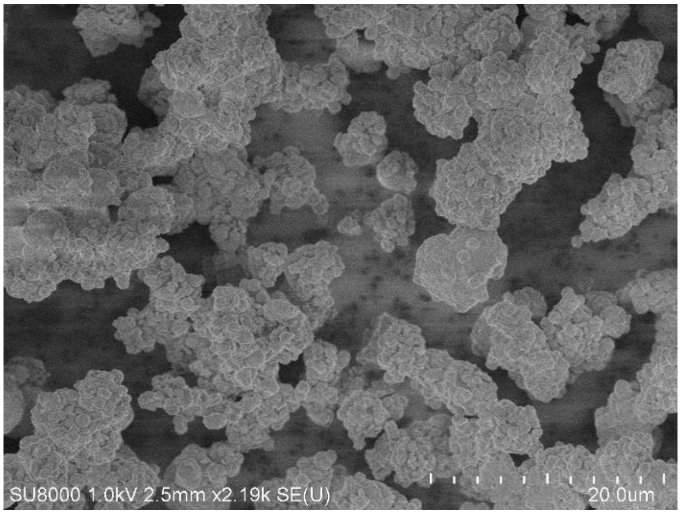

Figure S5. FESEM image of product obtained from OCRMs at low magnification

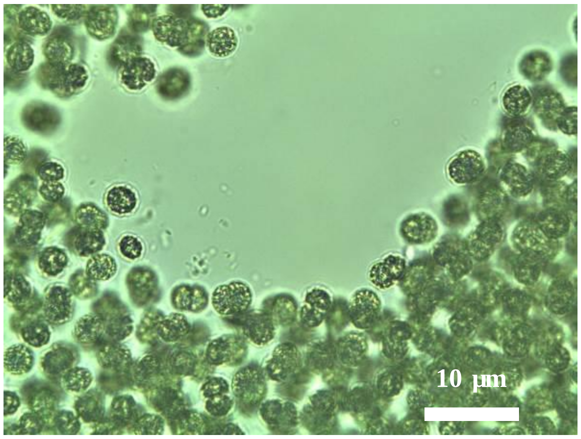

Figure S6. the OM image of cyanobacteria used in the experiment
